# The correlation between tumor size, lymph node status, distant metastases and mortality in rectal cancer patients without neoadjuvant therapy

**DOI:** 10.7150/jca.52165

**Published:** 2021-01-15

**Authors:** Dakui Luo, Zezhi Shan, Qi Liu, Sanjun Cai, Yanlei Ma, Qingguo Li, Xinxiang Li

**Affiliations:** 1 Department of Colorectal Surgery, Fudan University Shanghai Cancer Center, Shanghai 200032, China.; 2 Department of Oncology, Shanghai Medical College, Fudan University, Shanghai 200032, China.

**Keywords:** tumor size, lymph node status, distant metastases, rectal cancer, SEER

## Abstract

Tumor size has an effect on decision making for the treatment rectal cancer. Transanal local excision can be selected to remove rectal cancer with favorable histopathological features. It is generally recognized that the risk of lymph node involvement and distant metastases increases as the tumor enlarges. However, the majority of the studies classified patients into two groups using concrete value as a cutoff point. The coarse classification was not sufficient to reveal a correlation between the tumor size and lymph node status or distant metastases across the full range of sizes examined. Between 1988 and 2015, a total of 77,746 patients were diagnosed with first primary rectal cancer who had not received neoadjuvant therapy. These subjects were identified using the Surveillance, Epidemiology and End Results (SEER) database. The association between tumor size, lymph node status, distant metastases and cancer-specific mortality was investigated. Tumor size was examined as a continuous (1-30 mm) and categorical variable (11 size groups; 10-mm intervals). A non-linear correlation between increasing tumor size and the prevalence of lymph node involvement was observed, while a near-positive correlation between tumor size and distant metastases was presented. In addition, the 5-year and 10-year rates of rectal cancer-specific mortality were increased as the tumor enlarged. For small tumors (under 30 mm), a positive correlation was noted between tumor size and lymph node involvement. The clinical value of the tumor size should be reevaluated by exact classification.

## Introduction

Rectal cancer (RC) is the 8^th^ most frequently diagnosed cancer and the 10^th^ leading cause of cancer related deaths worldwide in 2018 1. Lymph node involvement and distant metastases have indicated poor prognosis in RC. It is believed that the risk of developing lymph nodal or distant metastases depends on intrinsic biological and tumor size characteristics since the larger tumor can readily metastasize 2, 3. Based on this theory, clinical guidelines recommend that transanal local excision can be adopted to remove lesions with favorable histopathological features, such as <3 cm size, T1, grade I or II, absence of lymphatic or venous invasion, or negative margins 4, 5. It is reasonable to assume that that a <3 cm tumor size with favorable histopathological features will be associated with low risk of lymph node involvement and distant metastases.

The tumor-node-metastasis (TNM) staging system is widely applied for prognostic prediction of colorectal cancer (CRC). However, the tumor size has not been included in the TNM staging system and previous studies did not reach a consensus regarding the prognostic value of the tumor size in CRC 6-10. Notably, these studies classified patients into two groups using concrete value (3 cm, 4 cm, 5 cm) as a cutoff point. The coarse classification interfered with the detailed effects of tumor size on lymph node status and distant metastases across the full range of sizes.

In the present study, we aimed to reveal the associations between tumor size and the risk of metastases (both lymph nodes and distant sites) in rectal cancer patients who did not receive neoadjuvant therapy across the size range of 1-100 mm using the Surveillance, Epidemiology and End Results (SEER) database. In addition, the association between tumor size and rectal cancer-specific mortality was evaluated.

## Material and methods

A total of 77,746 patients diagnosed with first primary rectal cancer who had not received neoadjuvant therapy were identified using the SEER database (1988-2015). In general, the inclusion criteria were detailed as follows: RC was the sole type of primary cancer; patients with definite tumor size were included; no neoadjuvant radiotherapy was administered; surgery was performed; detailed information regarding cancer-specific survival (CSS) and survival duration was included.

The following variables were included: age, gender, marital status, race, year of diagnosis, tumor size, grade, histology codes, T stage, N stage, M stage and survival information. The patients were classified into 11 categories according to primary tumor size (10-mm intervals, 1-100 mm and >100 mm). In addition, tumor size was evaluated as a continuous variable (1-30 mm). CSS was defined as the time from diagnosis to death resulting from RC.

The Kaplan-Meier method was used to estimate the actual rates of rectal cancer-specific mortality at 5 and 10 years. All statistical analyses were performed with SPSS 25.0 and the data were presented using GraphPad Prism 8.

## Results

The baseline characteristics of RC patients are summarized in Table 1. A total of 57,356 (73.8%) patients exhibited tumors that were smaller than 50 mm in size, whereas 19,415 (25.0%) patients exhibited tumors that were between 50 and 100 mm in size and 975 (1.2%) patients exhibited tumors that were larger than 100 mm in size. A total of 19,543 (25.1%) patients experienced lymph node involvement and 46,580 (58.6%) patients presented with lymph node-negative metastases. A total of 9,315 (12.0%) patients were classified as stage IV disease cases and 67,755 (87.1%) patients exhibited no evidence of distant metastases. By the end of the follow-up period, 25,813 (33.2%) patients did not survive due to RC.

**Figure 1:**
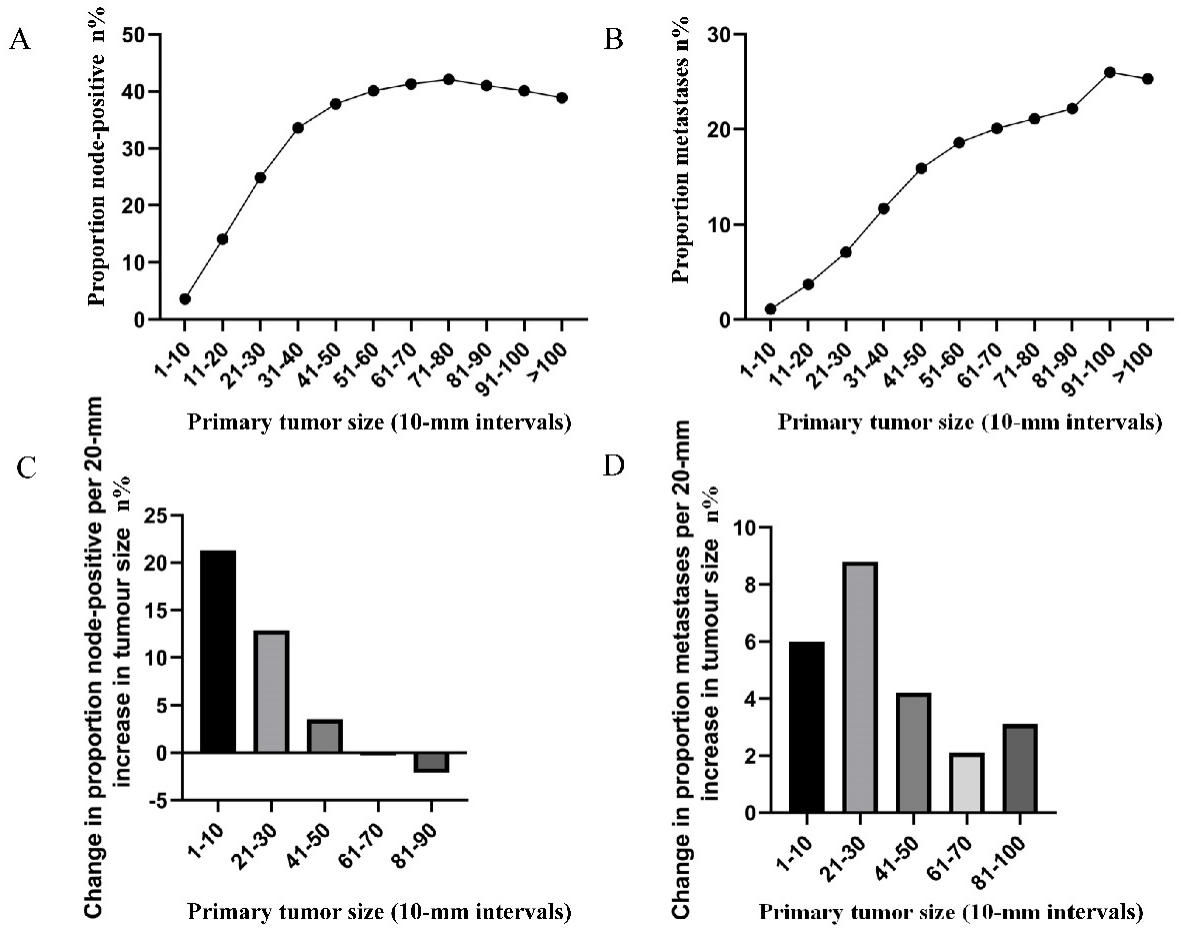
A. Prevalence of lymph node involvement at diagnosis among rectal cancer patients according to primary tumor size by 10-mm intervals. B. Prevalence of distant metastases at diagnosis among rectal cancer patients according to primary tumor size by 10-mm intervals. C. Increase in the prevalence of lymph node involvement per 20-mm increase in primary tumor size. D. Increase in the prevalence of distant metastases per 20-mm increase in primary tumor size.

The correlation between tumor size (in 10-mm intervals) and the probability of lymph node involvement in patients with definite lymph node status is presented in Figure 1A. A non-linear correlation between increasing tumor size and the prevalence of lymph node involvement was observed. The proportion of lymph node involvement elevated stepwise as the tumor size was enlarged between group 1 (1-10 mm) and group 5 (41-50 mm), while the escalating trend tended to be horizontal between group 6 (51-60 mm) and group 8 (71-80 mm). It is interesting to note that the proportion of lymph node involvement was decreased stepwise as the tumor size was enlarged between group 8 (71-80 mm) and group 11 (>100 mm). Subsequently, the association between tumor size (in 10-mm intervals) and the probability of distant metastases was investigated in patients with definite disease stage. As shown (Figure 1B), a near-positive correlation between tumor size and distant metastases was found. The proportion of distant metastases increased continuously from 1.1% for tumors that were 1-10 mm in size to 26.0% for tumors that were 91-100 mm in size. Furthermore, the absolute growth in the prevalence of lymph node involvement and distant metastases, as the tumor size was enlarged (per 20-mm), was also plotted (Figure 1C, 1D).

**Figure 2:**
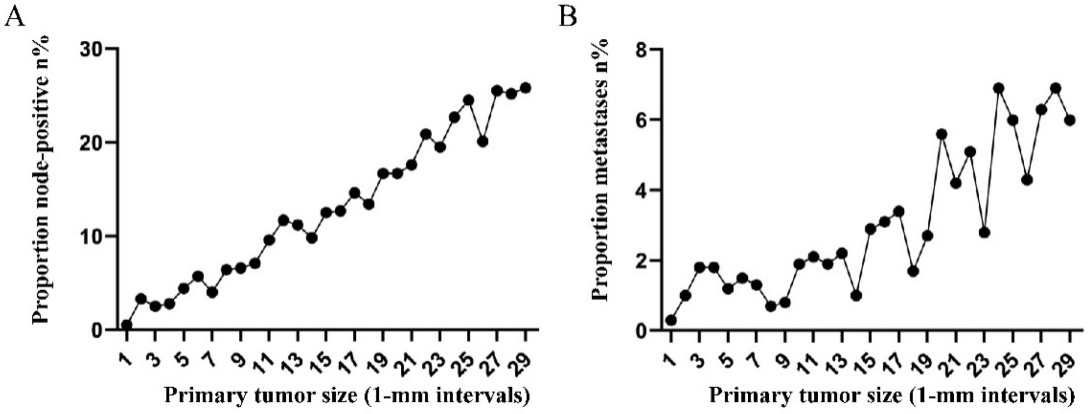
A. Relationship between lymph node involvement and primary tumor size among rectal cancer patients according to tumor diameter by 1-mm intervals (1-30 mm). B. Relationship between distant metastases and primary tumor size among rectal cancer patients according to tumor diameter by 1-mm intervals (1-30 mm).

To highlight the variation tendency of the association between tumor size and lymph node status and that of distant metastases in patients who had tumors smaller than 30 mm in size, the tumor size was examined as a continuous variable (1-30 mm). A near-positive correlation was noted between tumor size and lymph node involvement (Figure 2A). However, a small correlation between tumor size and distant metastases was noted (Figure 2B). The overall trend was increasing (Figure 2B).

**Figure 3:**
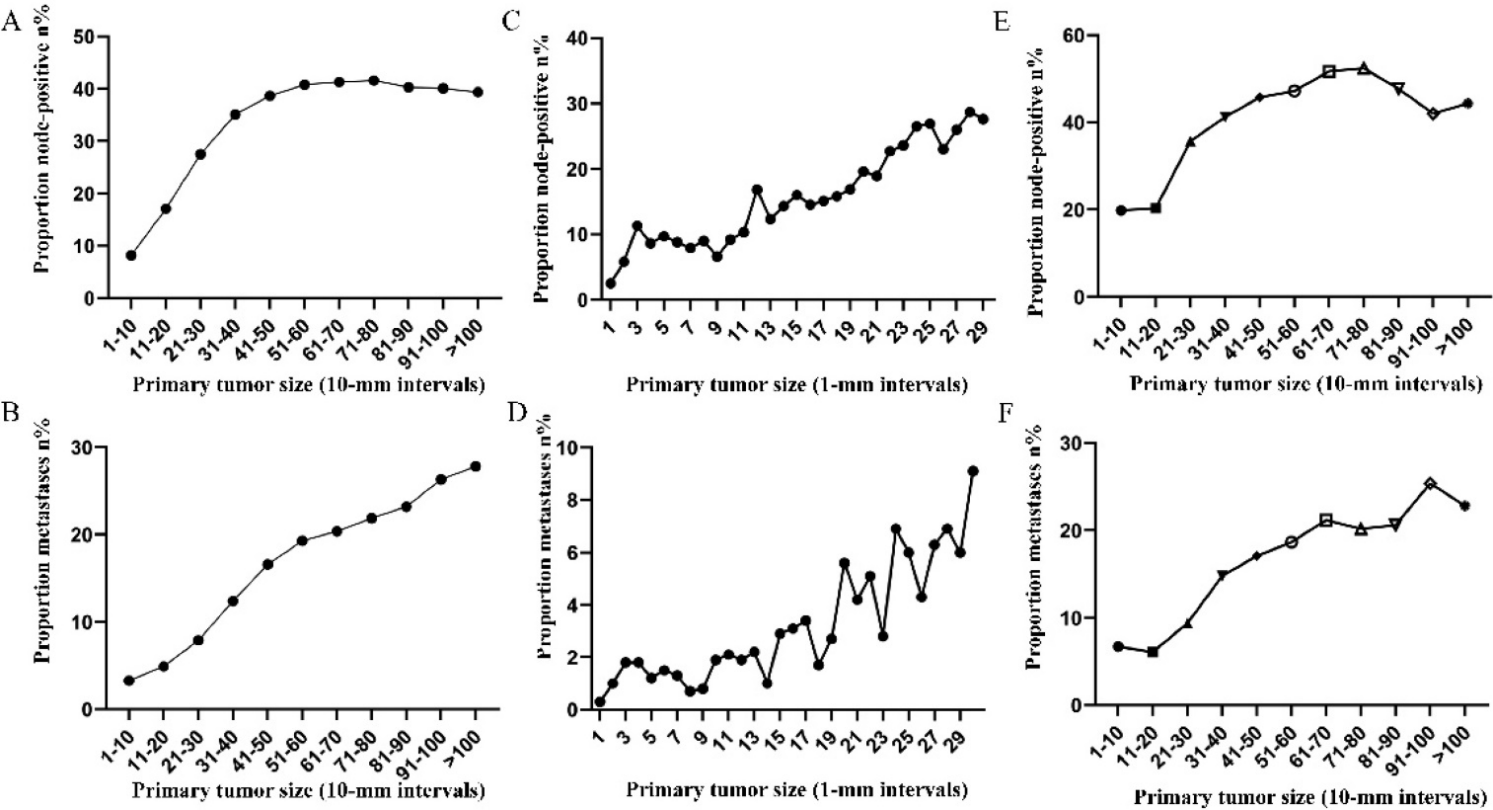
A. Prevalence of lymph node involvement at diagnosis among patients with rectal adenocarcinoma according to primary tumor size by 10-mm intervals. B. Prevalence of distant metastases at diagnosis among patients with rectal adenocarcinoma according to primary tumor size by 10-mm intervals. C. Relationship between lymph node involvement and primary tumor size among patients with rectal adenocarcinoma according to tumor diameter by 1-mm intervals (1-30 mm). D. Relationship between distant metastases and primary tumor size among patients with rectal adenocarcinoma according to tumor diameter by 1-mm intervals (1-30 mm). E. Prevalence of lymph node involvement at diagnosis among rectal cancer patients with mucinous adenocarcinoma according to primary tumor size by 10-mm intervals. F. Prevalence of distant metastases at diagnosis among rectal cancer patients with mucinous adenocarcinoma according to primary tumor size by 10-mm intervals.

**Figure 4:**
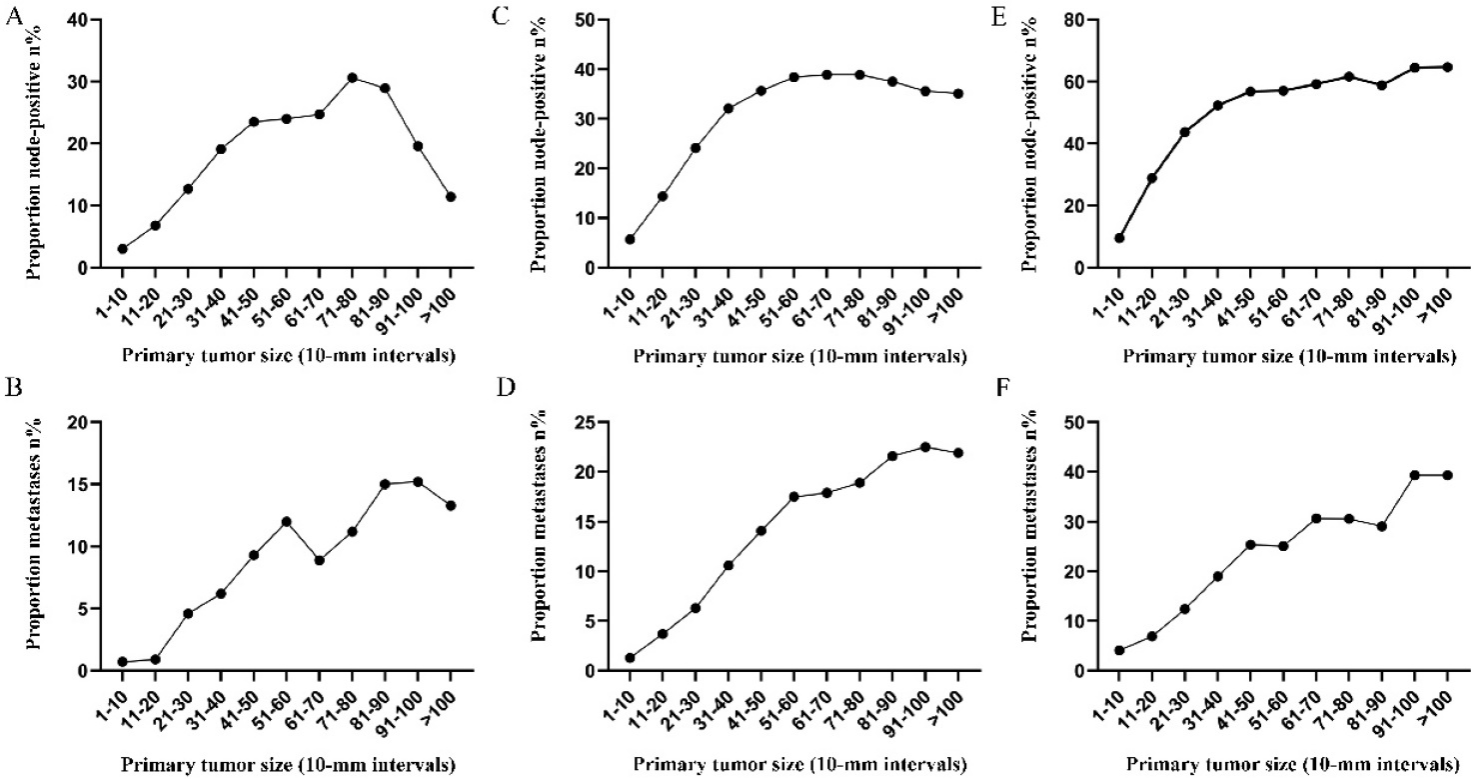
Prevalence of lymph node involvement or distant metastases at diagnosis among rectal cancer patients according to primary tumor size by 10-mm intervals based on different differentiation. A. Relationship between lymph node involvement and tumor size for patients with well differentiation. B. Relationship between distant metastases and tumor size for patients with well differentiation. C. Relationship between lymph node involvement and tumor size for patients with moderate differentiation. D. Relationship between distant metastases and tumor size for patients with moderate differentiation. E. Relationship between lymph node involvement and tumor size for patients with poor or undifferentiated differentiation. F. Relationship between distant metastases and tumor size for patients with poor or undifferentiated differentiation.

**Figure 5:**
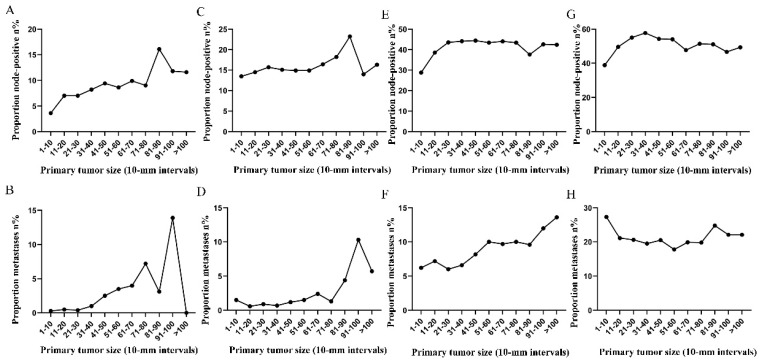
Prevalence of lymph node involvement or distant metastases at diagnosis among rectal cancer patients according to primary tumor size by 10-mm intervals based on different T stage. A. Relationship between lymph node involvement and tumor size for T1 patients. B. Relationship between distant metastases and tumor size for T1 patients. C. Relationship between lymph node involvement and tumor size for T2 patients. D. Relationship between distant metastases and tumor size for T2 patients. E. Relationship between lymph node involvement and tumor size for T3 patients. F. Relationship between distant metastases and tumor size for T3 patients. G. Relationship between lymph node involvement and tumor size for T4 patients. H. Relationship between distant metastases and tumor size for T4 patients.

Subsequently, the association between primary tumor size, the prevalence of lymph node involvement and distant metastases was examined for rectal patients stratified according to histological type, differentiation and T stage. For patients with adenocarcinoma, the association between tumor size (10-mm intervals or 1-mm intervals between 1-30 mm) and the prevalence of lymph node metastases was similar for the entire cohort. However, a positive correlation between tumor size (10-mm intervals) and distant metastases was more profound compared with that noted in the entire cohort (Figure 3A, 3B). As tumor size was examined as a continuous variable (1-30 mm), the overall trend was irregular (Figure 3C, 3D). A non-linear correlation between increasing tumor size, the prevalence of lymph node involvement and distant metastases was observed for patients with mucinous adenocarcinoma (Figure 3C, 3D). The proportion of lymph node involvement was increased as the tumor size was enlarged between group 1 (1-10 mm) and group 8 (71-80 mm), whereas the proportion was decreased between group 8 (71-80 mm) and group 10 (91-100 mm). Due to the limited sample size of patients with mucinous adenocarcinoma, tumor size was not examined as a continuous variable for this subgroup. The proportion of lymph node involvement was increased stepwise as the tumor size was increased between group 1 (1-10 mm) and group 8 (71-80 mm) for patients with well differentiated tumors, while this proportion was decreased sharply between group 8 (71-80 mm) and group 11 (>100 mm). The trend of lymph node involvement was similar to that noted for the entire cohort for patients with moderate differentiation. The proportion of lymph node involvement was increased stepwise as the tumor size was enlarged for patients with poor differentiation between group 1 (1-10 mm) and group 11 (>100 mm). However, between group 5 (41-50 mm) and group 11 (>100 mm), the trend tended to be horizontal. The association between tumor size and distant metastases was also examined and only patients with moderate differentiation presented a significantly positive correlation. Between group 5 (41-50 mm) and group 11 (>100 mm), the prevalence of distant metastases was fluctuated in patients with well or poor differentiation (Figure 4). Generally, tumor size represented horizontal growth index, while T stage reflected vertical infiltration index. Subsequently, we evaluated the association between tumor size and lymph node status as well as that between tumor size and distant metastases according to the different T stage of the tumors. A minimal correlation was evident between tumor size and lymph node involvement or distant metastases. However, the overall trend was indicative of an association between T1, T2 and lymph node involvement and between T1, T2, T3 and distant metastases (Figure 5). The increase noted in the association trend was relative to the higher tumor stage.

**Figure 6:**
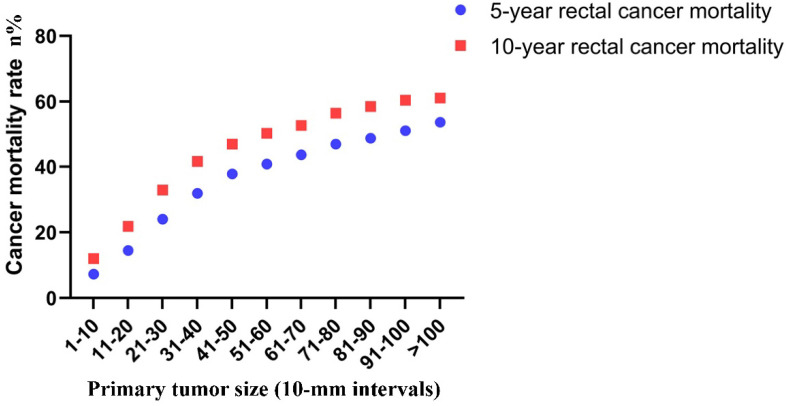
Actuarial 5-year and 10-year rates of rectal cancer-specific mortality among all rectal cancer patients according to the size of the primary tumor by 10-mm intervals.

Finally, the correlation between tumor size and risk of rectal cancer-specific mortality was investigated. The 5-year mortality increased stepwise from 7.3% for tumors that were 1-10 mm in size to 53.6% for tumors that were >100 mm in size. The 10-year mortality increased stepwise from 12.0% for tumors that were 1-10 mm in size to 61.1% for tumors that were >100 mm in size (Figure 6).

## Discussion

The tumor, lymph node, metastasis (TNM) staging system has been established as the most important prognostic factor in rectal cancer. In addition, tumor deposits, serum CEA levels, tumor regression score, circumferential resection margins, lymph vascular invasion, perineural invasion, microsatellite instability and RAS and BRAF mutations should also be considered in the prognostic prediction and treatment decision making 11. However, tumor size was excluded from the prognostic factors. In general, the T stage represented vertical tumor penetration across the bowel wall, whereas the tumor size reflected the horizontal growth index. Evidence regarding the prognostic value of the tumor size is limited and fails to reach a definitive conclusion.

Several studies have shown that tumor size did not present any prognostic impact on colorectal cancer patients 12-14. However, the results have been contradictory over the last years. Tayyab et al. 15 demonstrated a direct association between tumor volume and overall survival in rectal cancer. Kornprat et al. established tumor size as an independent prognostic parameter for patients with colorectal cancer. The authors of this study found that the optimal cut-off values were dependent on different parts of the large bowel 10. Brunner et al. 16 demonstrated that tumor size was a predictor for regional lymph node metastasis in T1 rectal cancer using the SEER database. It is interesting to note that Takahashi et al. highlighted that tumor size was associated with tumor recurrence in colon cancer instead of rectal cancer 17. Our previous study demonstrated that the mortality risk of node positivity increased as tumor enlarged until a threshold tumor size (tumor size of 7-8 cm) was reached in colon cancer. The value of tumor size in rectal cancer should not been neglected.

In the present study, we examined the correlation between tumor size, lymph node status, distant metastases and mortality in a cohort of 77,746 rectal cancer patients without neoadjuvant therapy. Tumor size was examined as a continuous (1-30 mm) and categorical variable (11 size groups; 10-mm intervals) instead of previous coarse classification. A linear correlation was found between tumor size and the risk of lymph node involvement for tumors of group 1 (1-10 mm) and group 5 (41-50 mm). For relatively large tumors (higher than 50 mm), a notable departure was observed. The probability of a lesion being lymph node positive was 42.1% and reached the highest level for a tumor size of 71-80 mm. When the association between tumor size and lymph node involvement was examined for patients with small tumors (less than 30 mm) stratified in 1-mm intervals, an upward trend was noted as tumor size increased from 1 to 29 mm (from 0.5 to 25.8%). The indications of local excision for rectal cancer should be applied with caution. Chen et al. identified a tumor size of <5 cm as a strong negative prognostic factor for local recurrence in rectal adenocarcinoma 9. However, the authors of that study failed to identify tumor size as an independent predictor of lymph node involvement. In the present study, a near-positive correlation between tumor size and distant metastases was found. However, the overall trend was increasing. The prevalence of distant metastases at diagnosis increased gradually from 1.1% for tumors 1-10 mm in size to 25.3% for tumors larger than 100 mm in size. This phenomenon may have occured due to the small sample size noted in each subgroup. It was also shown that distant metastasis was an early event during tumor progression. The tumors will acquire higher potential to metastasize as their growth is increased 18. Similarly, the 5-year mortality increased stepwise from 7.3% for tumors 1-10 mm in size to 53.6% for tumors >100 mm in size. The majority of the previous studies grouped all tumors into two sets using concrete value as a cutoff point. We can fully understand the association between tumor size and lymph node status, distant metastases and mortality by refining the tumor size spectrum.

In the present study, the data indicated that the probability of group 6 (tumor size of 51-60 mm in diameter at diagnosis) being node-positive was equal to the probability of group 10 (tumor size of 91-100 mm in diameter at diagnosis) being node-positive. These data suggested that the probability of developing extra lymph node metastases was extremely low during the period in which a tumor grew from 51-60 mm to 91-100 mm. In contrast to these observations, when a lesion had grown from 1-10 mm to 11-20 mm, the probability of developing new lymph node metastases was 10.5%. Subsequently, it was found that the proportion of distant metastases and the 5-year mortality were increased stepwise as the tumor size was enlarged between groups 6 and 10. We speculated that the increase in the 5-year mortality resulted from the increasing risk of distant metastases instead of lymph node metastases between groups 6 and 10.

Subgroup analysis revealed a positive correlation between tumor size and distant metastases, which seemed to be higher in adenocarcinoma cases compared with the entire cohort, while a non-linear correlation between increased tumor size, the prevalence of lymph node involvement and distant metastases was observed in mucinous adenocarcinoma cases. One explanation for the lack of a tumor size effect on lymph node and distant metastases for mucinous adenocarcinoma is its high malignant potential and heterogeneity.

In summary, we observed a non-linear correlation between tumor size and the prevalence of lymph node involvement and a near-positive correlation between tumor size and distant metastases using a large sample of rectal cancer patients. The shapes of the curves presented slight variation for the different subgroups. The clinical value of tumor size should be reevaluated by exact classification.

## Figures and Tables

**Table 1:** Characteristics of rectal cancer patients with definite record of tumor size and without receiving neoadjuvant therapy in SEER (1988-2015)

Characteristic	Value	Number of patients (%)
Age at diagnosis (years)	<60	24392 (31.4%)
	≥60	53354 (68.6%)
Gender	Male	43375 (55.8%)
	Female	34371 (44.2%)
Marital status at diagnosis	Married	45824 (58.9%)
	Unmarried	29005 (37.3%)
	Unknown	2917 (3.8%)
Race	White	64278 (82.7%)
	Black	5875 (7.6%)
	Other/Unknown	7593 (9.8%)
Year of diagnosis	1988-2003	45565 (58.6%)
	2004-2015	32181 (41.4%)
Tumour size (mm)	1-10	7074 (9.1%)
	11-20	8329 (10.7%)
	21-30	12965 (16.7%)
	31-40	15274 (19.6%)
	41-50	13714 (17.6%)
	51-60	9012 (11.6%)
	61-70	5247 (6.7%)
	71-80	2878 (3.7%)
	81-90	1377 (1.8%)
	91-100	901 (1.2%)
	>100	975 (1.3%)
T stage	T1	16130 (20.8%)
	T2	16100 (20.7%)
	T3	33409 (43.0%)
	T4	5872 (7.6%)
	Unknown	6235 (8.0%)
Lymph node-status	Negative	46580 (58.6%)
	Positive	19543 (25.1%)
	Unknown	11623 (14.9%)
Distant metastases	No	67755 (87.1%)
	Yes	9315 (12.0%)
	Unknown	676 (0.9%)
Histological type	Adenocarcinoma	56499 (72.7%)
	Mucinous adenocarcinoma	4403 (5.7%)
	Signet ring cell carcinoma	382 (0.5%)
	Others	16462 (21.2%)
Differentiation	Well	6404 (8.2%)
	Moderate	53859 (69.3%)
	Poor	10933 (14.1%)
	Undifferentiated	918 (0.9%)
	Unknown	5832 (7.5%)
Death from rectal cancer	No	51933 (66.8%)
	Yes	25813 (33.2%)
